# Inhibition of p38 mitogen-activated protein kinases may attenuate scar proliferation after cleft lip surgery in rabbits via Smads signaling pathway

**DOI:** 10.1186/s40001-022-00757-1

**Published:** 2022-07-20

**Authors:** Qian Ding, Jin Yue, Ling-fa Xue, Yao-xiang Xu, Wen-lin Xiao

**Affiliations:** 1 Department of Stomatology, the Affiliated Hospital of Qingdao University, No. 16, Jiangsu Road, Qingdao, 266003 China; 2 School of Stomatology, Qingdao University, Qingdao, 266071 Shandong China

**Keywords:** Cleft lip, p38MAPK, p-Smad2, p-Smad3, Scar

## Abstract

**Background:**

Cleft lip repair surgery always results in visible scarring. It has been proved that scar formation can be reduced by inhibiting the p38 mitogen-activated protein kinases (p38MAPKs) signaling pathway. However, the interaction between p38MAPK and Smads in scar formation is still controversial.

**Methods:**

This study was designed to investigate whether inhibition of p38MAPK reduces postoperative scar formation of cleft lips on rabbits via the Smads signaling pathway. Scar models in rabbits after cleft lip surgery were created and their fibroblasts were extracted. Then the expression of p38MAPK was disturbed by adenovirus in vitro and Vivo. The scar thickness was measured and scar tissues were excised for Sirius red staining and immunohistochemistry to detect the expression of type I collagen (col I), type III collagen (col III), and α-smooth muscle actin (α-SMA). The underlying mechanisms of p38MAPK knockdown on the extracellular matrix and Smad signaling pathway were invested in vitro using the EdU assay, Western blot, RT PCR, and immunofluorescence.

**Results:**

p38MAPK knockdown suppresses the expression of p-smad3 and p-smad2 in fibroblasts, modulating the expression of its target genes, such as α-SMA, col I, and col III. When Ad-P38MAPK-1 was injected into lip scar, it reduced the expression of scar-related genes and scar thickness when compared to the negative control groups.

**Conclusions:**

In rabbits, inhibiting p38MAPK expression prevents scar proliferation through inhibiting the Smad signaling pathway after cleft lip surgery.

## Introduction

In China, the prevalence of cleft lip and palate was about 1.5‰ ~ 2‰. The degree of scar hyperplasia after cleft lip surgery was positively correlated with anxiety [1]. The prevention and treatment of scar hyperplasia have been of great interest in cleft lip operations, and have been addressed by various means ranging from surgical suture materials to postoperative laser treatment [2–4]. However, current therapeutic measures were inadequate to resolve skin wound scar hyperplasia. Kim et al. found that Asian populations are predisposed to scar formation [5, 6]. The rate of scar hyperplasia formation in Asian patients was as high as 36.3%, significantly higher than other races [7]. Scar hyperplasia was more obvious in wounds with high levels of mechanical force, such as the cause of the respiration and cross-joint wounds after chest surgery, while reducing mechanical force could significantly reduce scar hyperplasia in wounds [8, 9]. Because of lip movement, the cleft lip surgery wound was subjected to a high level of tension stress. Mammalian p38MAPKs are activated by a wide range of cellular stresses as well as in response to inflammatory cytokines, which then influence the cell cycle or cytoskeleton remodeling [10]. Furthermore, it has not only the basic function of MAPK but also mechanical properties [11, 12].

In vitro and in vivo, inhibition of the p38MAPK signaling pathway has shown so far to reduce scar formation and the expression of related factors [13–15].

The interaction of p38MAPK and Smads in scar formation is still controversial [13, 14, 16, 17]. This study was designed to investigate whether inhibition of p38MAPK expression may play a specific role in reducing scar hyperplasia via the Smads signaling pathway during scar formation after cleft lip surgery in rabbits.

## Materials and methods

### Laboratory animals

Thirty healthy male New Zealand white rabbits from Qingdao Kanga Biological Technology Company, weighing between 2.0 and 2.5 kg were used. The rabbits were all free of infection and had normal upper clefts. The Ethics Committee of the Affiliated Hospital of Qingdao University, Qingdao, China approved all protocols.

### Laboratory reagents, instruments, and consumables

Reagents included p38MAPK antibody (Novus, CO, USA), Smad2 antibody (Affinity Biosciences Cat# AF6449, RRID:AB_2835272), Smad3 antibody (Affinity Biosciences Cat# AF6362, RRID:AB_2835210), phospho-Smad3 antibody (Affinity Biosciences Cat# AF3365, RRID: AB_2834780), phospho-Smad2 antibody (Santa Cruz, CA, USA). SDS–PAGE electrophoresis reagents and transmembrane apparatus were from Bio-Rad, and a Tanon 5200 Chemiluminescent detection system was used (Tanon, Shanghai, China). Real-time PCR was carried out using SYBR Premix Ex Taq (Dye fluorescence quantitative kit, Takara, Japan), Prime scrip RT reagent Kit with gDNA Eraser (Takara, Japan), RNAiso Plus (Takara, Japan), and Step OnePlus fluorescence quantitative PCR instrument (ABI, CA, USA). Besides, laboratory equipment included an optical microscope, an imaging system (Nikon, Japan), and a microsyringe (Hamilton, Switzerland).

### Methods

#### Animal experiment and grouping

Thirty rabbits were divided into three groups of ten each: blank control, Ad-NC, and Ad-P38MAPK-1 group. Cleft lip repair was performed on all of rabbits. Scarring scar after cleft lip surgery in rabbits was created using the methods described in our previous articles [18]. The first and second weeks after the operation, the Ad-NC group was injected with adenovirus without the interference sequence and the Ad-P38MAPK-1 group was injected with adenovirus p38MAPK interference sequence. The injection volume was 40 μL, and the density was 3 × 10^10^ pfu/mL. The blank control group was not injected with any reagent. Two weeks after the second injection, the rabbits were anesthetized and their scar tissue was obtained.

#### Construction of the recombination adenovirus vector

The cDNA sequence of the rabbit full-length p38MAPK gene was obtained from GenBank. Then, three shRNA sequences were designed (Table 1). shRNA expression vectors were constructed by ligating annealed complementary shRNA oligonucleotides into the pDC316–shRNA–ZsGreen vector (Oligobio BioTechnology, Beijing, Co., Ltd.) following the manufacturer’s instructions. The negative control shRNA was constructed in the same way. Plasmid DNA was extracted from E. coli DH5a transformants using a Plasmid Mini Kit (Promega, Madison, WI) following the manufacturer’s instructions. The insertion of the desired oligonucleotide sequences into the vector was verified by restriction analysis and sequencing. Three recombinant plasmids vectors were constructed and named pDC316–ZsGreen–shRNA-1, -2, and -3. Then, pDC316–ZsGreen–shRNA vectors were, respectively, linearized and used to transfect HEK293A cells to produce adenovirus particles named Ad-P38MAPK-1, -2, and -3. The negative control vector Ad-P38MAPK-scrumble was also constructed. HEK293A cells were infected with diluted viruses and cultured in an incubator (Heraeus Holding GmbH, Hanau, Germany) at 37 ºC and 5% CO_2_ for 48 h before being observed under a fluorescence microscope. Using a hole-by-hole dilution titer, the number of fluorescent cells decreased with the increase of the dilution multiple. The conversion formula was as follows: MOI = viral titer (PFU/mL) × the volume of the virus solution (mL)/the number of cells.

**Table 1:** shRNA DNA oligo sequences of p38MAPK

p38MAPK-1-F p38MAPK-1-R	5ʹ-GCCTGACCTATGATGAAGTTTCAAGAGAACTTCATCATAGGTCAGGCTTTTTT-3ʹ 5ʹ-GATCAAAAAAGCCTGACCTATGATGAAGTTCTCTTGAAACTTCATCATAGGTCAGGC-3ʹ
p38MAPK-2-F p38MAPK-2-R	5ʹ-GCGGCTACTCAAACATATGATTCAAGAGATCATATGTTTGAGTAGCCGTTTTTT-3ʹ 5ʹ-GATCAAAAAACGGCTACTCAAACATATGATCTCTTGAATCATATGTTTGAGTAGCCGC-3ʹ
p38MAPK-3-F p38MAPK-3-R	5ʹ-GGCAGATCTGAACAACATTTTCAAGAGAAATGTTGTTCAGATCTGCCTTTTTT-3ʹ 5ʹ-GATCAAAAAAGGCAGATCTGAACAACATTTCTCTTGAAAATGTTGTTCAGATCTGCC-3ʹ

#### Measurement of the thickness of lip scar tissue

Four weeks after surgery, the scar thickness of the upper lip of all rabbits was measured using Vernier calipers. The scar’s midpoint was used as the measuring point.

#### Sirius red staining

All specimens were rinsed in descending order of density. Specimens were immersed in xylol I and xylol II for 20 min each, dipped in absolute ethyl alcohol I and ethyl alcohol II for 10 min each, then cleaned in an ethanol gradient (95% to 70%) for 5 min. Sirius red staining (Servicebio Technology, Wuhan, China) was used to examine pathological changes and the expression of collagen fibers. Tissue blocks were dehydrated in an ascending ethanol series for 5 min before being cleared in xylol and embedded in neutral balata. Light microscopy was used to examine the specimens, which were then analyzed by technicians who were blind to the groups to which the specimens belonged.

#### Immunohistochemical staining

Samples were incubated in primary antibodies of α-SMA (Cell Signaling Technology, America) overnight at 4 °C. Corresponding secondary antibodies were added for 50 min incubations at 4 °C after washing with PBS three times. Then, 60 mL of DAB solution was added after washing with PBS 3 times. The tissue blocks were flushed with distilled water, dyed with hematoxylin, dehydrated with an ethanol gradient, cleared in xylol, and then fixed in neutral balata. Finally, three experimental pathologists who were blinded to the identity of the groups analyzed the images.

#### Primary culture of rabbit lip scar fibroblasts

A rabbit cleft lip scar model was established, and the scarred skin was harvested 3 weeks after the operation. The harvested skin scar tissue was immersed in an ice-cold amortization medium (0.95% HBSS, 0.238% HEPES, 0.6% D-glucose, 0.035% NaHCO3, and penicillin/streptomycin 100 U/mL) (Sigma-Aldrich, St. Louis, MO) for approximately 30 min. Then, a tissue block was taken out with tweezers and subcutaneous fat was scraped with a surgical blade. The tissue was washed several times with PBS and cut into pieces about 1 mm^3^. The tissue was digested with collagenase at room temperature for 45 min with continuous oscillation. The scar fibroblasts of rabbit lips were resuspended and cultured in DMEM/F12 medium (Sigma-Aldrich). The medium was discarded, cells were washed with PBS, and 1–2 mL trypsin solution was added. The cells were then centrifuged at 1000 rpm for 3 min, and the supernatant was discarded. Complete medium DMEM/F12 was added for culturing and passage.

#### Immunofluorescence assay

Fibroblasts seeded on coverslips (Solarbio, TC coverslip 14 mm, China) and transfected vectors for 48 h were fixed in 0.4% paraformaldehyde for 1 h. After being washed three times with PBS for 3 min each fibroblasts were permeabilized in 0.3% Triton X-100 for 15 min. Then the washing procedure was repeated and fibroblasts were blocked with 5% Normal Goat Serum (Solarbio, SL038) for 1 h at room temperature. After that, cells were incubated with primary antibody p-smad3 (1:100) overnight at 4 ℃. The following day, the fibroblasts were washed again with PBS before being incubated with secondary antibody (1:200) for 1 h at room temperature in the dark. Then, a drop of DAPI(Beyotime, China)was added to the glass slide, the cell slide was placed on the DAPI, and the edge was sealed with nail polish. IF images were captured with a laser scanning confocal microscope (Leica TCS SPE, Germany) and an inverted fluorescence microscope (EVOS fl auto, America). The antibodies used are as follows: vimentin (5741 T, Cell Signaling Technology), p-smad3 (AF8315, AffinitY), goat anti-rabbit IgG-FITC (abs20004ss, absin), anti-rabbit IgG (H + L), F(ab')2 Fragment (Alexa Fluor^®^ 555 Conjugate) (4413 s, Cell Signaling Technology).

#### Cell proliferation assays

The fibroblasts were seeded in a six-well plate at a density of 5–10*10^4/mL, and wells were divided into a blank group, a negative control group, and an experimental group. The following day, the negative control group and the experimental group were treated with Ad-P38MAPK-scramble and Ad-P38MAPK-1, respectively. After 48 h, the procedures were carried out according to BeyoClick™ EdU Cell Proliferation Kit with Alexa Fluor 594(Beyotime China). Fibroblasts were detected under an inverted fluorescence microscope. The percentage of proliferating cells was determined by counting EdU-positive cells and total cells in 10 randomly captured fields per well.

#### Quantitative real-time PCR

RNA was extracted from scar tissues and fibroblasts using RNAiso Plus (TaKaRa, Japan), chloroform, isopropyl alcohol 75% ethanol, and DEPC The primeScript RT reagent kit with a gDNA eraser (TaKaRa, Japan) was then used to generate cDNA from RNA. After that, quantitative real-time PCR assays were performed using the Step OnePlus fluorescence quantitative PCR instrument and standardized by GAPDH. The 2 − ΔΔCt method was used to calculate the relative expression levels. Primers used in the PCR assay are listed in Table 2.

**Table 2:** Primers used in quantitative real-time PCR

Gene	Primer Sequence (5′–3′)	Annealing Temperature, °C(60℃)	Product size, bp
Col I	F:AGATGGCTACCCAACTTGCC	60	340
	R:GGGCCAACGTCCACATAGAA		
Col III	F:AACAATGGTAGTCCTGGCGG	60	154
	R:CACCGTTCTTACCGGGTTCA		
α-SMA	F:TGTCAGGAATCCCGTGAAGC	60	73
	R:CATTGTCACACACAAGGGCG		
p38MAPK	F:GTATGCTGGGTTGGGTGTGC	60	220
	R:GCGTCACCAGATACACGTCA		

#### Western blot analysis

NP-40 lysis buffer (Solarbio, China), PMSF(Solarbio, China) ,and Protease Inhibitor Cocktail(Solarbio, China)were used to extract proteins. Total proteins were transferred to PDVF membranes (GE, America) from 10% SDS–PAGE after determining the protein content (BCA Protein Colorimetric Assay Kit, Elabscience, China). Membranes were incubated in primary antibodies α-SMA (1:1000), Smad2 (1: 2000), Smad3 (1: 1000), phospho-Smad2 (1: 5000), phospho-Smad3 (1: 2000), and p38MAPK (1: 500) at 4 °C overnight. After washing, membranes were incubated with secondary antibodies (Rabbit anti-Goat IgG–HRP, abisin, China at room temperature for 2 h. Chemiluminescence was performed with Thermo ECL (elabscience, China and visualization was done with a Tanon gel imager. We used the Image J software to quantify using β-actin as an internal standard. This experiment was repeated three times to obtain average values.

### Statistical methods

In total, each experiment was performed three times. All data are presented as mean ± SD, and significant differences were determined using Student's *t* tests or one-way ANOVA. p < 0.05 was considered significant.

## Results

### The scar model of the cleft lip was successfully constructed and the scar fibroblasts were extracted

**Fig. 1:**
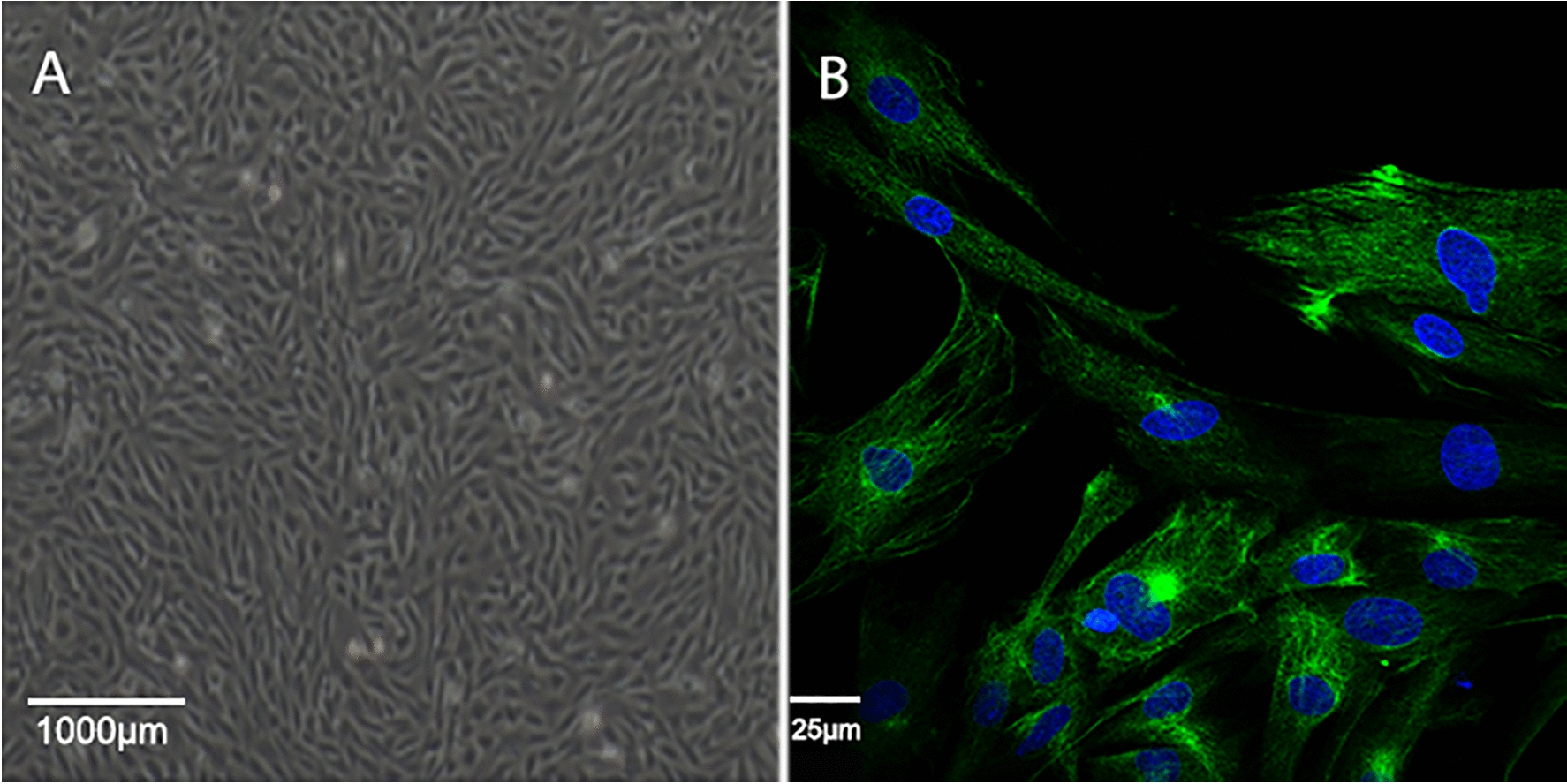
Microscopic observation of rabbit lip scar fibroblasts. A representative image of primary cultured rabbit lip scar fibroblasts (400×) is shown. Fluorescence images of cells stained against vimentin (green) to identify fibroblasts. Nuclei were dyed with DAPI (blue) (630×)

The cells appeared elongated or flattened, with some triangular in shape. During the growth process, cells arranged themselves in radial, braided, or spiral (Fig. 1A). Besides, immunofluorescence detection showed that a positive reaction of diffuse cytoplasmic fluorescence with an anti-vimentin antibody (Fig. 1B).

### The recombinant adenovirus vector was successfully constructed and its knockout efficiency was up to 67%

**Fig. 2:**
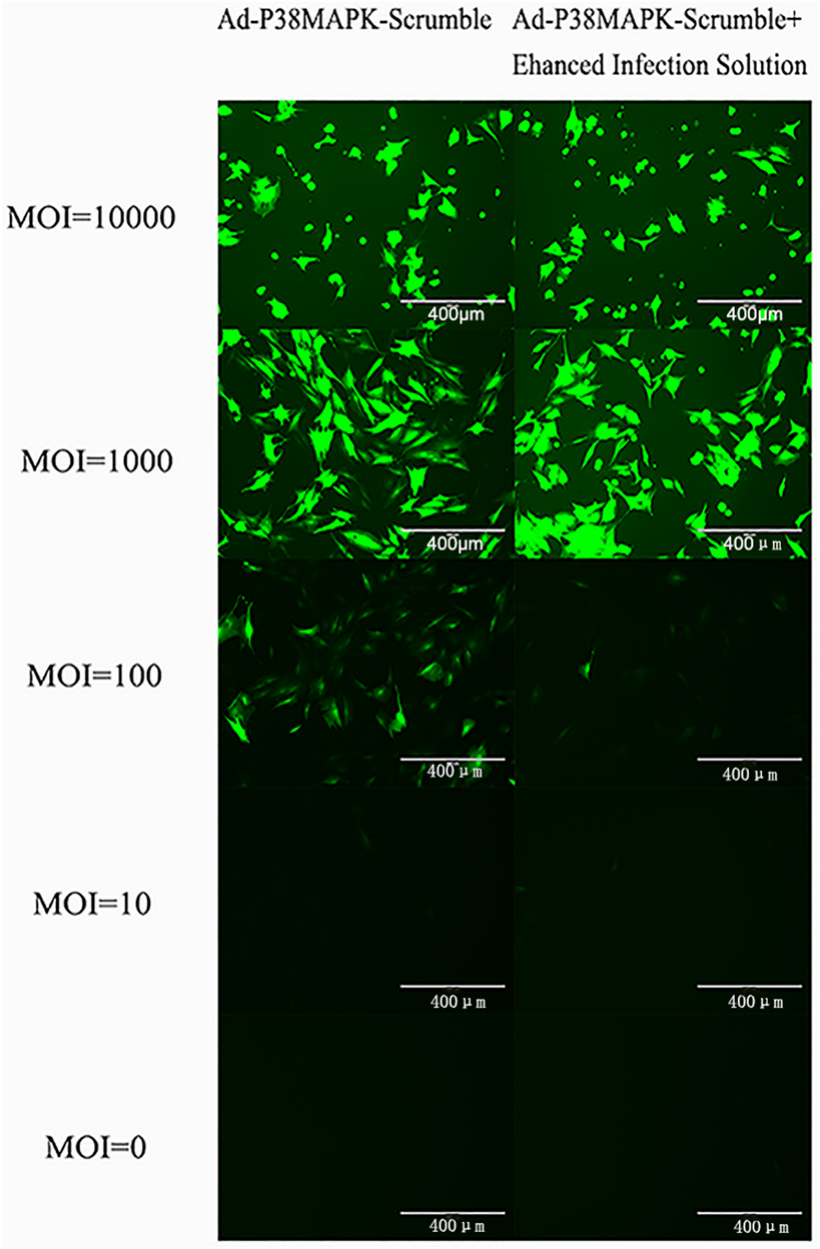
Fluorescence microscopy after rabbit lip scar fibroblasts were transfected by Ad-P38MAPK-scrumble after 48 h (400×)

**Fig. 3:**
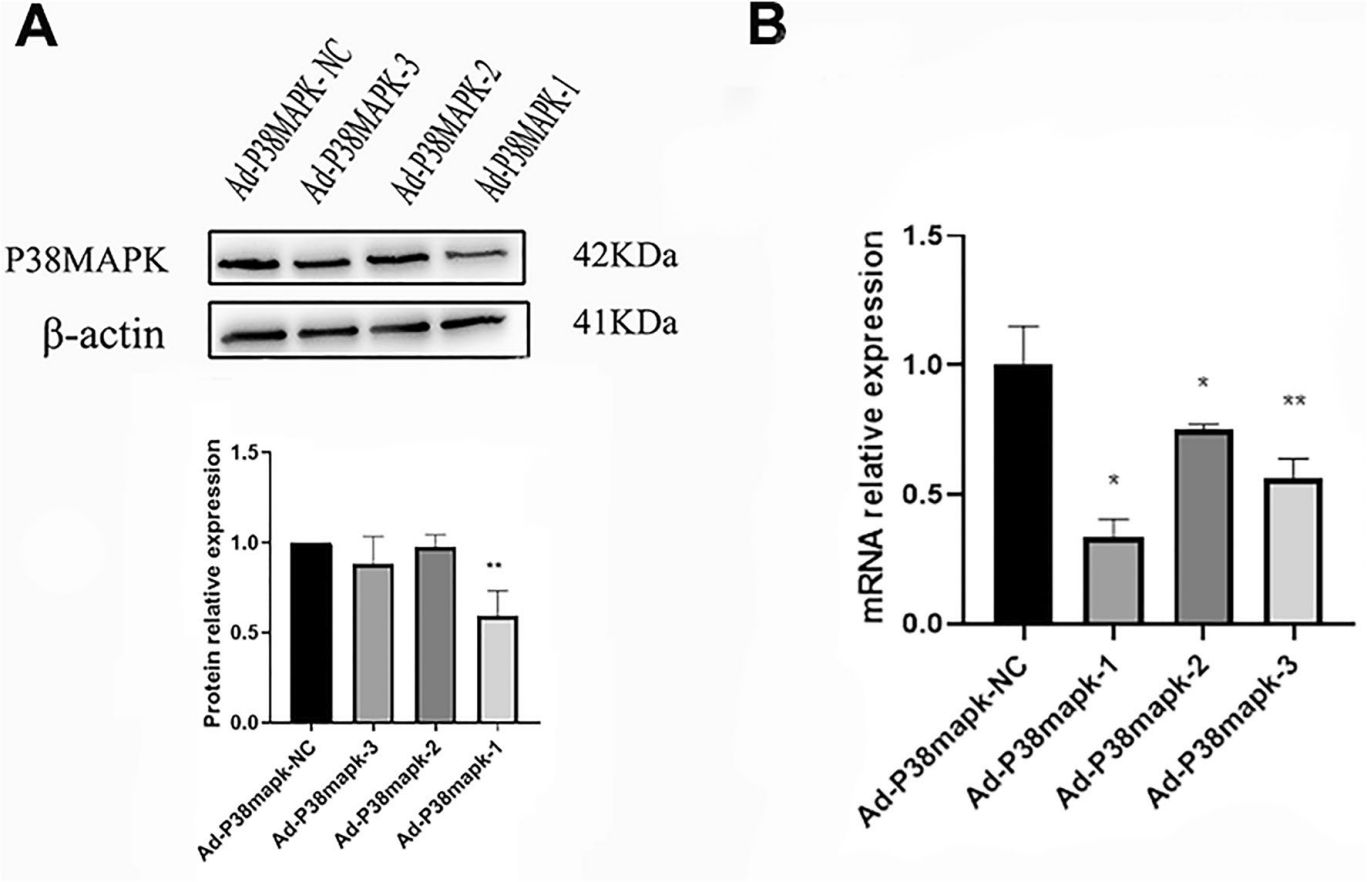
Real-time PCR and western blot analysis of p38MAPK mRNA and protein levels in primary cultured rabbit lip scar fibroblasts infected with different adenoviruses for 48 h. **A** Represents western blot results. **B** Represents real-time PCR results. Results are presented as the mean ± S.D, * *p* < 0.05 and **p < 0.01

The transfection efficiency can reach more than 99% when the MOI is set to 100 (Fig. 2). Real-time PCR was used to determine p38MAPK mRNA expression levels after Ad-P38MAPK-1, -2, and -3 were used to transfect scar fibroblasts for 48 h. The results showed that, when Ad-P38MAPK-1 was transfected, the mRNA expression level of p38MAPK was significantly lower than when Ad-P38MAPK-2 and -3 were transfected. p38MAPK protein expression was suppressed in rabbit lip scar fibroblasts at 48 h after Ad-P38MAPK-1, -2, and -3 transfection. The protein expression level of p38MAPK was downregulated significantly by Ad-P38MAPK-1 transfection (*p* < 0.01) (Fig. 3A). Both real-time PCR and western blot analyses indicated that Ad-P38MAPK-1 had the highest interference efficiency, reaching nearly 67% (Fig. 3A, B). Thus, the Ad-P38MAPK-1 was chosen for further research.

### p38MAPK knockdown significantly reduced scar thickness

**Fig. 4:**
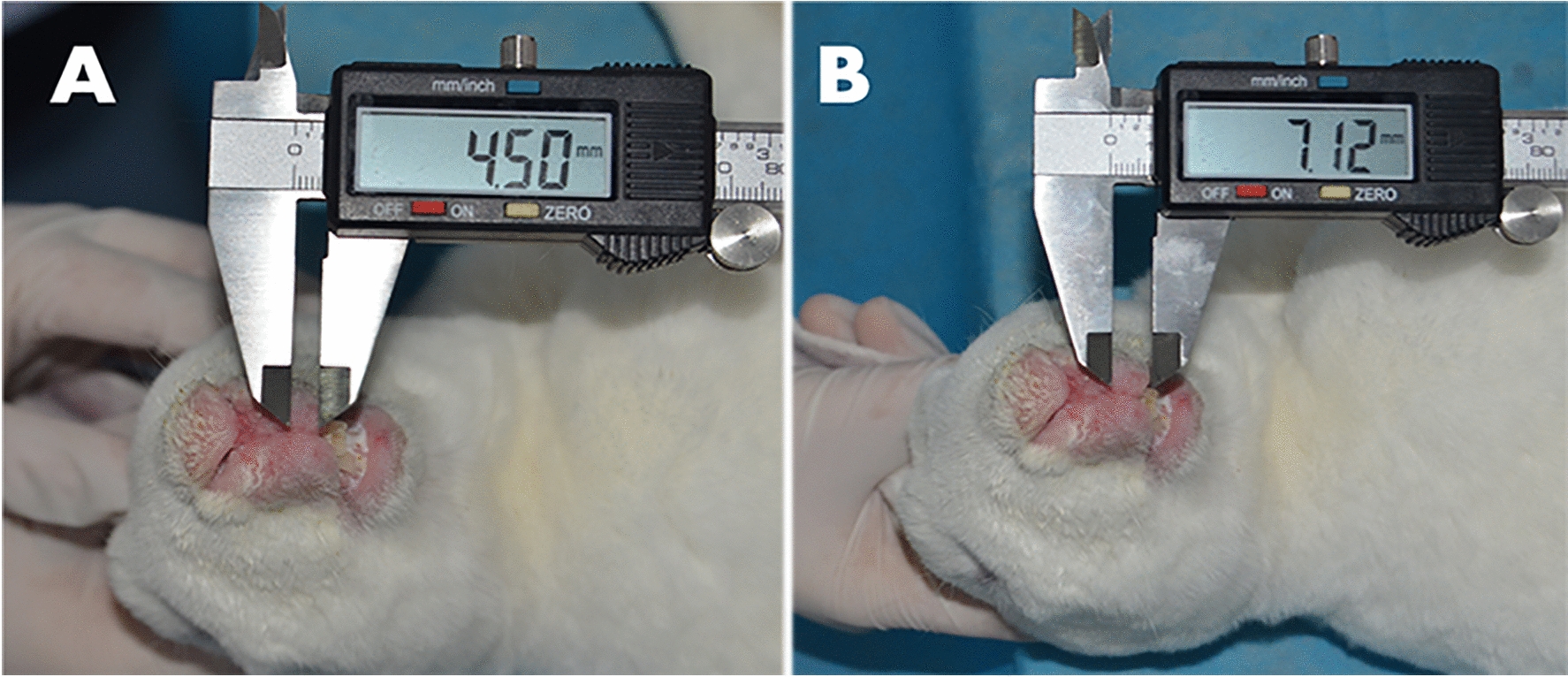
Four weeks after surgery for the cleft lip, the average thickness of upper lip scar was about 4 mm in the surgical group (**A**), and about 7 mm in the blank and negative control groups (**B**)

Four weeks after cleft lip surgery, the average thickness of upper lip scar in the surgery group injected with adenovirus p38MAPK interference sequence was about 4 mm (Fig. 4A), while the average thickness of upper lip scar in the blank control and the Ad-NC groups injected with the adenovirus without the p38MAPK interference sequence was about 7 mm (Fig. 4B). The results showed that adenoviruses that silence the p38MAPK gene had a therapeutic effect on scar hyperplasia after upper lip surgery in rabbits.

### *p38MAPK knockdown changes the expression of Col I, col III, and α-SMA *in vivo* and vitro*

**Fig. 5:**
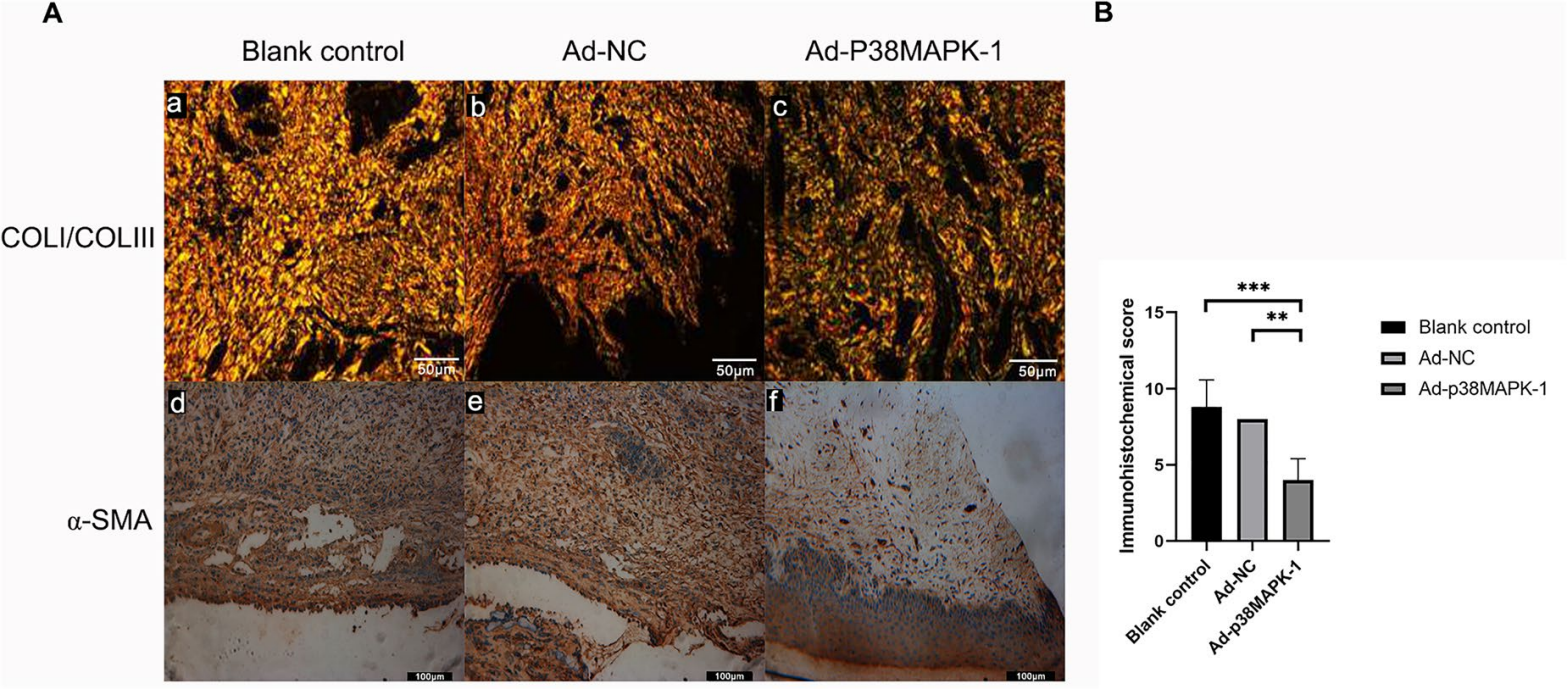
Effect of p38MAPK knockdown on the extracellular matrix in vivo. A. Sirius red staining for col I (green) and col III (brown) of different groups 7 days after infection (200×). Expression changes of α-SMA following p38MAPK knockdown as detected by immunohistochemistry 14 days after infection, Scale bar = 100 µm. B. Immunohistochemical scoring of α-SMA, ** *p* < 0.01 and ***p < 0.001

**Fig. 6:**
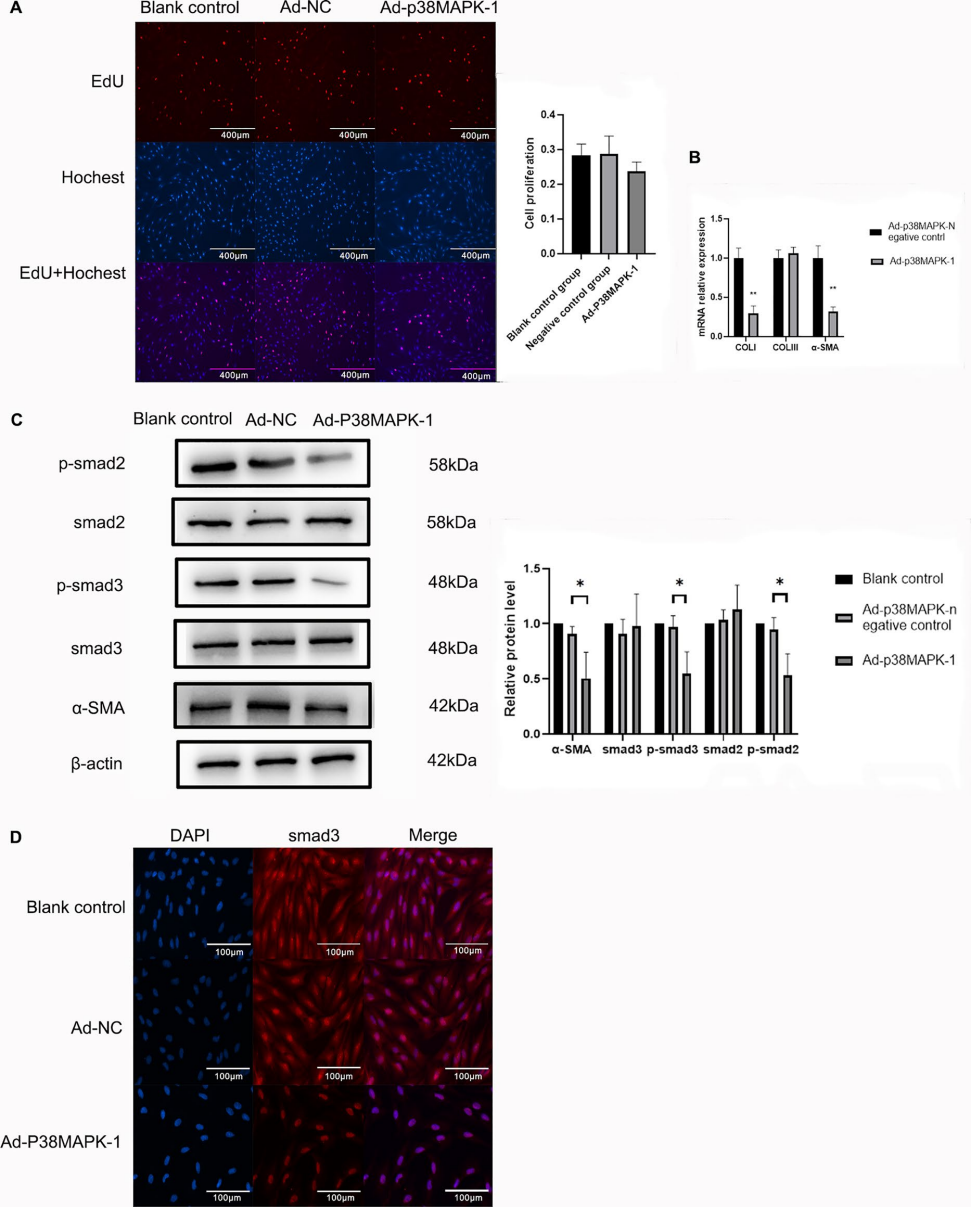
Effects of p38MAPK knockdown on extracellular matrix and Smads signaling pathway in fibroblast. **A** Effect of p38MAPK on the proliferation of scar fibroblasts was examined using the EdU assay. **B** mRNA expression levels of col I, col III, and α-SMA following p38MAPK knockdown as detected by RT-PCR. **C** Changes in protein expression of smad2, p-smad2, smad3, p-smad3, and α-SMA following p38MAPK knockdown as detected via western blotting. **D** Changes in protein expression of p-smad3 following p38MAPK knockdown as detected via immunofluorescence assay. Results are presented as the mean ± S.D, * *p* < 0.05

Following p38MARK knockdown, we observed the expression of col I and col III in the scar tissue of the rabbits’ upper lips. After viral transfection, the content of col I declined markedly, while the content of col III increased markedly after viral transfection at 7 days after the operation according to the Sirius red staining results. After 1 week, the tissue had a high concentration of col III and a low concentration of col I. (Fig. 5A). In addition, immunohistochemical results showed that the expression of α-SMA was significantly lower in the Ad-MAPK-1 group (Fig. 5A, 5B) than that in the control groups (Fig. 5A) at 14 days after injection. PCR results showed that col and α-SMA expression levels in the Ad-P38MAPK-1 group were significantly lower than those in the control group 48 h after virus transfection in fibroblast (Fig. 6B).

### Effects of p38MAPK on scar fibroblast proliferation and the Smads signaling pathway

The results of the western blot reveal that p38MAPK knockdown inhibits the expression of α-SMA, p-smad3, and p-smad2 (Fig. 6C). In addition, this effect is not achieved by inhibiting cell proliferation (Fig. 6A). The results were statistically significant (*p* < 0.05) (Fig. 6C). Immunofluorescence results also demonstrated that the expression level of p-smad3 was depressed by the p38MAPK knockdown (Fig. 6D).

## Discussion

The skin on the human body is very sensitive to mechanical signals [19]. Improper mechanical force can have a significant impact on wound healing and fibrosis [20]. Mechanical force can cause the differentiation of fibroblasts (FBs) into myofibroblasts (MFBs), which increases the expression of α-smooth muscle actin (α-SMA, a marker of differentiation of MFBs) and increases the extracellular matrix (ECM) component in the wound. This proved that wound fibrosis can be regulated by the mechanical force of FB [21]. It was indicated that the mechanism of wound healing after cleft lip treatment under the influence of external mechanical tension may be different from the wound healing mechanism affected by the nonexternal tension of the human body.

The p38MAPK signaling pathway, a new protein isoform, affiliates with the MAPK family. p38MAPK stimulates the production of collagen and fibronectin in the extracellular matrix of human lung scar FBs [22]. The p38MAPK activity was inhibited in the mouse scar test, affecting collagen and fibronectin production in the subconjunctival sac [13]. In the early stages, we used the upper lip hypertrophic scar tissues from patients who had cleft lip surgery to study the in vitro culture of scar FBs. Periodic tension has been shown to increase the expressions of a-SMA and p38MAPK, which are positively correlated with the tensile force. p38MAPK blockers can reduce the expression of p38MAPK, and the expression of a-SMA also decreases. The findings demonstrated that the p38MAPK signaling pathway was involved in the differentiation of hypertrophic scar fibroblasts mediated by tension stress.

The Smads family is a highly conserved molecule in eukaryotic cells and is widely found in nematodes, fruit flies, and mammals. Studies have shown that excessive activation of the Smads signaling pathway induces excessive proliferation of FBs [23]. When the Smads family members Smad2 and Smad3, are phosphorylated, these transcription factors bind their binding partners. This Smads complex is then shuttled into the nucleus, where it activates transcription of MFB genes, including SMA, calponin, and collagen during scar formation [24–26]. Several studies have found that the expression of Smad2 and Smad3 increases during the scar formation process of in various tissues [27–29].

Many studies have confirmed the interaction of p38MAPK and Smads in scar formation. According to one study, the p38MAPK inhibitor significantly disrupted the Smad2/3/4 complex formation and thus decreased the invasiveness of keloid FBs [16]. Moreover, the p38MAPK pathway interfered with Smads expression and was able to reverse Smads suppression in keloid FBs [16]. The accumulation of evidence suggests that the cross-talk between the Smad-dependent pathway and Smads independent mitogenic pathway drives normal fibroblast cellular mechanisms toward aggressive, tumor-like characteristics of keloid FB [30]. Woeller et al. support the hypothesis that salinomycin targets the Smads pathway indirectly via the p38MAPK pathway. The p38MAPK pathway contains a feedforward loop that stimulates Smad2/3 phosphorylation and activation [17].

Studies on retinal pigment epithelial cells have shown that the Smads signal is dependent on p38MAPK to regulate the expression of ColI and fibronectin in scar extracellular matrix [13]. However, studies on fibroblasts conjunctival scar showed that the inhibition of p38MAPK phosphorylation did not lead to an increase of ColI and fibronectin through Smads signal [14]. These are contradictory conclusions. Yamanaka et al. attributed the existence of cross-talk between Smads and p38MAPK signaling pathways to different cell types [14]. Whether p38MAPK regulates scar proliferation via Smads after cleft lip surgery is currently unclear.

Long et al. found that SB203580 suppresses the expression of contractile genes in vascular smooth muscle cells. Surprisingly, siMAPK14 promotes these genes in vascular smooth muscle cells. They speculated that the inhibitor may affect other signaling pathways at a high concentration [31]. As a result, the recombinant adenovirus vector carrying a foreign gene was our first choice.

Our previous studies have observed the hyperplasia trend of scar after the cleft lip surgery in a rabbit animal model, and determine the timepoint of the highest proliferation degree of scar after cleft lip surgery. The results showed scars shrunk and volumes reduced 3 to 4 weeks after surgery, indicating that scar proliferation is most severe 3–4 weeks after surgery [18]. Therefore, in this study, we chose the fourth week after the surgery to observe the effect of p38MAPK adenovirus on scar proliferation.

This study demonstrated that the expression of phospho-p38MAPK protein and its mRNA decreased, and the expression of phospho-Smad2 and phospho-Smad3 protein and its mRNA also decreased 2 weeks after the injection of p38MAPK gene silencing adenovirus into the cleft lip of rabbits. Then, we explored its mechanism in scar fibroblasts. It was suggested that the inhibition of p38MAPK expression also affects the expression of Smad2 and Smad3 in scar fibroblasts. The inhibition of p38MAPK expression may play a role in inhibiting the scar proliferation via the Smads signaling pathway after cleft lip surgery in rabbits.

Recombinant adenoviral vectors can be used in variety of applications. Oncolytic viruses, genetic vaccines, and gene therapy vectors are a few examples. Gendicine, a recombinant human p53 adenovirus, was approved for use in the treatment of head and neck cancer in 2003 and has demonstrated a high level of safety in clinical application records [32] Using recombinant adenovirus vectors to reduce scarring on the lip is promising. In the future, we will look into how p38MAPK affects scarring in other areas of the maxillofacial region, such as scarring from cleft palate repair surgery. In addition, fibrosis is also an important promoter in wound healing, and inhibiting the p38MAPK pathway early may affect wound healing [33]. It is important to determine the optimal treatment time and injection dose.

## Conclusions

Finally, this study looks into the antifibrotic effect of silencing p38MAPKs in a rabbit model and scar fibroblasts. Our findings show that inhibiting p38MAPKs reduce scar formation via suppressing the expression of smad3 and smad2 in rabbit lip scar fibroblasts.

## Data Availability

All data generated or analysed during this study are included in this published article.

## Abbreviations

p38MAPK: p38 mitogen-activated protein kinases
α-SMA: α-Smooth muscle actin
COL I: Type I collagen
COL III: Type III collagen
